# Optimized Analog Multi-Band Carrierless Amplitude and Phase Modulation for Visible Light Communication-Based Internet of Things Systems

**DOI:** 10.3390/s21072537

**Published:** 2021-04-05

**Authors:** Luis Rodrigues, Mónica Figueiredo, Luis Nero Alves

**Affiliations:** 1 Department of Electronics, Telecommunications and Informatics, University of Aveiro, 3810-193 Aveiro, Portugal; nero@ua.pt; 2 Instituto de Telecomunicações, Universidade de Aveiro, Campus Universitário de Santiago, 3810-193 Aveiro, Portugal; monica.figueiredo@ipleiria.pt; 3 School of Technology and Management, Polytechnic of Leiria, 2411-901 Leiria, Portugal

**Keywords:** visible light communications, Internet of Things, m-CAP modulation

## Abstract

This paper presents a multi-user Visible Light Communication (VLC)-based Internet of Things (IoT) system using multi band-Carrierless Amplitude and Phase (m-CAP) modulation for IoT applications. The proposed system uses a digital m-CAP modulator embedded in a ceiling LED light fixture and analog receivers, aiming at low-cost, low-power, and small-sized IoT devices. The performance was evaluated in terms of the filtering stage design and the usage of guard bands. Different pairs of emitter and receiver filters were considered. While Bessel and Butterworth analog filters were tested in the analog receiver, the digital m-CAP modulator pulse shaping filter considered raised cosine filters, as well as digital matched filters for the analog Bessel and Butterworth filters. Regarding the guard bands, two approaches were considered: either by using the raised cosine roll-off factor (bandwidth compression) or by suppressing the even bands. The Bit Error Rate (BER) performance was obtained by simulation. The usage of the Bessel filter in the receiver, along with a digital matched filter, proved to be the best solution, achieving a BER lower than $10^{-3}$ for an Eb/No of 6 dB, using a third-order filter. Furthermore, guard bands should be used in order to mitigate inter-band interference in order to have improved performance when multiple users intend to simultaneously communicate.

## 1. Introduction

Internet connection among everyday objects is expected to become ubiquitous in the next decade [1]. As the technologies evolve, more devices are expected to be deployed, which will pose several requirements for Internet of Things (IoT) devices. As opposed to radio frequency devices, Visible Light Communication (VLC) technologies have not been targeting the IoT requirements of low-cost, low-power, low-complexity, and small-sized devices. Since VLC systems often use a Field-Programmable Gate Array (FPGA) or a Digital Signal Processor (DSP) in their implementations, they are able to perform a fully digital signal processing. However, the cost, power consumption, and size of such implementations can be a significant impairment for their massive deployment. In this paper, we propose the usage of IoT devices with analog frontends, which aim for receiver simplicity in order to cope with the stringent IoT requirements.

Most of the IoT concept relies on widespread simple sensors and actuators, usually resorting to wireless communications. This is commonly known as Wireless Sensors Networks (WSNs), and it has become a growing research topic, having a wide range of applications, which are gradually being adopted in IoT systems [2,3,4,5,6,7,8]. WSNs are characterized as a local network with two main types of devices: nodes and gateways. Network nodes perform a specific task, such as a sensor node (temperature, humidity, RFID tag [5], and body sensors [6]) or an actuator node (irrigation systems [9] and lighting system control). Such devices usually have low power consumption, low cost, and small-size requirements [10], making it impractical to have direct Internet access. Therefore, typical WSNs have a common gateway, which serves as the interface between the nodes and the Internet. Common network architectures include: (i) start networks (all the nodes are connected directly to one gateway); (ii) mesh networks (some nodes may act as data relays between devices and gateways); (iii) tree networks (intermediary data concentrators are used between nodes and gateways) [11]. Most of the current communication technologies in the IoT (WiFi, BLE, ZigBee, LoRaWAN, SigFox^®^, and NB-IoT) are able to operate in the star network architecture. Despite the lack of link redundancy, the most commonly deployed architecture is the star network due to the simplicity of the routing algorithms, which also reduces the device’s complexity [12]. In the context of VLC, such systems often use existing lighting infrastructure. Therefore, the star network topology can be easily implemented in VLC scenarios, where the IoT node devices communicate with the ceiling light fixtures, which act as gateways.

Although Radio Frequency (RF) is widely adopted for WSNs, its performance may degrade in some IoT scenarios due to the high device spatial density, resulting in an increased number of communication packet collisions [12]. Alternative communication technologies are thus welcome to address the lack of connectivity in some places and/or relieve the RF spectrum. During the last decade, visible light communication has been proposed as an alternative and/or complementary communication technology. Derived from Optical Wireless Communication (OWC), VLC exploits the visible part of the electromagnetic spectrum to communicate, resorting to a light source with modulation capabilities. Common VLC systems use Light Emitting Diodes (LEDs) since they allow having common infrastructures for illumination and communication. When compared to RF technology, VLC has several advantages, namely [13,14,15]: (i) a free/unregulated spectrum; (ii) the possibility of spectrum re-usage in indoor scenarios, since every room has all the spectrum available; (iii) a high data rate due to the large available bandwidth; (iv) the absence of electromagnetic interference; (v) improved security; and (vi) improved energy efficiency by combining communication and lighting systems. In indoor scenarios, VLC can potentially combine lighting and communication, as it can reuse the LED lighting systems, which already represent about 40–45% of the total lighting systems [16]. Other scenarios can also be considered. Underground mine sites are known for having issues with RF communications [17]. Already existent lighting system in mines could communicate with underground sensors/actuators. Furthermore, VLC may offer improved usage security in RF hazardous environments such as aircrafts [18,19] and highly flammable environments [20]. Due to lighting systems’ typical configuration, the light fixtures being in the ceiling illuminating downwards, the star network topology is naturally implemented in VLC systems: while node devices are spatially spread across a lit area, the ceiling light fixtures act as gateways, communicating both with the nodes and the Internet. Thus, a single ceiling gateway could simultaneously cover multiple users [21,22,23], with an increased probability of having a line-of-sight link.

IoT-targeted VLC devices and systems are currently very limited, and they are in an early development state. Some scenario examples that can be found in the literature include Intelligent Transportation Systems (ITSs) [24,25], Visible Light Positioning (VLP) [13], VLC smart labels [26,27], and initialization of IoT devices for security improvement [28], amongst others, such as indoor healthcare data transmission, infotainment services, drone-to-drone communications, and augmented/virtual reality, relying on both photodiode- and camera-based communications [29,30]. Although most proposed devices require significant processing resources, an effort to reduce their complexity and cost has also been pursued by some research teams, namely Corner Cube Retroreflectors (CCRs) (including battery-less devices) [31,32,33] and VLC backscattering architectures [34,35,36]. In [37], the authors proposed augmented spatial modulation to be used in VLC-based MIMO IoT scenarios, using modified Spatial Modulation (SM) to overcome the spatial identification problem for low complexity systems. Nevertheless, current developments in VLC systems are not targeting the IoT scenarios, where multiple users need to share the channel resources, along with the cost, power, and size of the IoT requirements, since they commonly consider a single user, or in the case of multiple-user architectures, they make use of complex modulations, which require powerful processing devices.

IoT systems, in particular WSNs, usually have multiple devices in a network, simultaneous requiring the transmission of independent data. This requirement implies the usage of a system architecture capable of multi-user communications. Different approaches have been proposed in the literature such as Time Division Multiple Access (TDMA), Optical Code Division Multiple Access (OCDMA), Wavelength Division Multiple Access (WDMA), and Space Division Multiple Access (SDMA) [38]. Depending on the application, such schemes may present some drawbacks: the TDMA scheme decreases the spectral efficiency and requires complex medium-access management, including time synchronism between network nodes; WDMA allows simultaneous medium access; however, it requires different wavelengths to be generated, which is not compatible with current LED lighting systems; SDMA requires the creation of several narrow-beam VLC channels, as well as the usage of sophisticated protocols for handling the handover; OCDMA offers the best compromise for IoT systems, since it does not require additional medium access control; however, additional demodulation processing needs to be taken into account for low-cost and low-power devices. Another common scheme used in multi-user VLC is Orthogonal Frequency Division Multiple Access (OFDMA) modulation, where a set of orthogonal bands is assigned to different users. Furthermore, Non-Orthogonal Multiple Access (NOMA) has been recently proposed for VLC systems, aiming at increasing the bandwidth in multiple access schemes by providing the entire channel bandwidth to all users. Power-Domain-NOMA (PD-NOMA) is the most common variant used in VLC, and it uses power allocation for each user: the greater the user distance is, the higher the transmitted power is. The decoding process is performed by applying successive interference cancellation, the data being recovered iteratively by the users closer to the emitter. However, both OFDMA and NOMA require a high processing power for signal demodulation in IoT devices, thus increasing the price and power consumption, which would not fit the IoT requirements [38,39,40]. Alternatively, multi band-Carrierless Amplitude and Phase (m-CAP) modulation has been proposed for VLC systems as it presents high spectral efficiency and easy implementation [41]. In an IoT context, m-CAP can be used to allow multiple access from different devices at the same time, assigning a set of bands per user. The concept was recently proposed, where m-CAP and Subcarrier Multiplexing (SCM) were combined, allowing up to 20 users in a single VLC system, with a total data rate of 162.5 Mb/s [42]. However, aiming at IoT scenarios, the node devices would require a digital implementation of an m-CAP demodulator, which could negatively impact the IoT device’s requirements.

This paper proposes an m-CAP modulated system, where the demodulation is performed resorting to analog circuitry. As opposed to a digital m-CAP demodulator, using an analog demodulation architecture could potentially demodulate m-CAP signals with minimal hardware, allowing low-cost, low-power, and small-sized devices [43]. A full analog implementation of CAP for optical systems was previously proposed in [44], making use of analog transversal filters for optical communications. Analog demodulation of a digitally generated m-CAP signal for visible light communications was first presented in [45]. Commonly found in the literature, the digital m-CAP modulation frequency spectrum lacks guard bands; although the continuous frequency spectrum presents higher spectral bandwidth, it poses severe requirements at the receiver. Typical m-CAP systems use a raised cosine filter as a pulse shaping filter. However, such filters are not feasible in the analog domain. Moreover, abrupt frequency response decay with analog filters is usually difficult to achieve due the components’ tolerances. Therefore, easy-to-implement analog filters, such as Bessel and Butterworth filters, should be considered at the receiver side. This paper studies the system’s performance regarding two parameters: filter choice and guard bands. The filter choice analysis evaluates the system’s performance regarding the analog filter at the receiver side, as well as the impact of substituting the raised cosine filter by one that matches the receiver filter. Concerning the guard band analysis, it evaluates the performance when guard bands are considered, either created by the suppression of the even bands, modulating only the odd bands, or alternatively, by varying the raised cosine filter roll-off factor, compressing the bandwidth of each band.

The paper is structured as follows: Section 2 describes the theoretical model for the proposed system. Section 3 describes the simulation setup. Section 4 presents the achieved results, and lastly, Section 4 states the conclusions of the paper.

## 2. System Description

**Figure 1:**
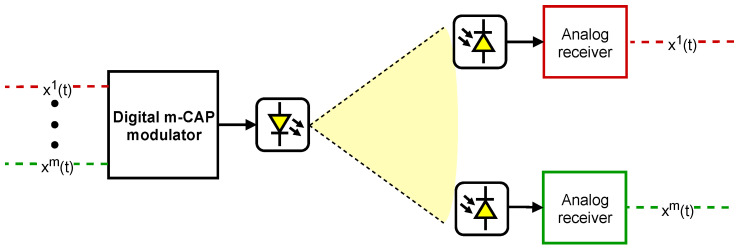
Proposed m-CAP VLC-based IoT system architecture using digital modulation and analog demodulation.

This section describes the proposed system in terms of its architecture and presents the theoretical models for the emitter and receiver, i.e., modulation and demodulation. The proposed system architecture is presented in Figure 1.

The system’s main components are the emitter and the receivers. The emitter is an m-CAP digital modulator, which aggregates *m* data sources into a modulated signal, where each data source is modulated into one band. The m-CAP signal is transmitted by a ceiling LED light fixture, being propagated in the VLC channel, assumed to be in a line-of-sight configuration. The optical signal is then captured by the IoT node devices. Each IoT node listens to a single band, and the demodulation is performed in the analog domain, resorting to a quadrature mixer and filtering, which will be detailed in Section 2.2.

**Figure 2:**
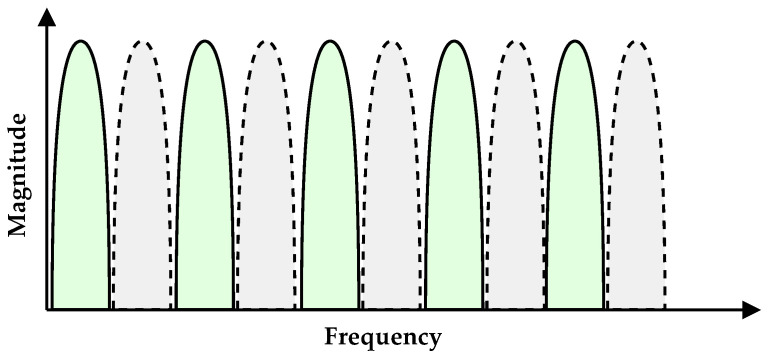
Modulated m-CAP signal frequency spectrum: gray/dashed bands may be suppressed to create guard bands.

Although the m-CAP frequency spectrum is typically contiguous, as in Figure 2, guard bands may be created by suppressing the even bands (gray/dashed), decreasing the interference between bands. Despite the spectral efficiency degradation being halved, the overall performance of individual bands will benefit from the same receiver filter specifications. This ensures simple implementations at the receiver side, which results in low-cost, low-power, and small-sized VLC-based IoT devices. As an alternative, when using a raised cosine filter in the emitter, the bandwidth of each band could be compressed by adjusting the filter roll-off factor. The relation between the roll-off factor and the guard bands will be discussed later in Section 2.3.2. Nevertheless, guard bands degrade the spectral efficiency, as well as the available orthogonal channels, limiting the maximum number of connected devices. Regarding the spectral efficiency, several IoT applications require a limited data rate. In fact, current RF technologies used in the IoT were designed with data rates as low as 100 bit/s, as in the case of SigFox^®^. Therefore, considering the available LED bandwidth, spectral efficiency degradation is the price to pay to meet the IoT requirements, allowing simple devices to operate in a multiple-user scenario. Concerning the maximum number of connected devices, m-CAP could be combined with TDM, such as the active bands in Figure 2 alternating in the time domain, in two distinct time slots: in the first time slot, the green bands would be active, and in the second, only the gray bands would be modulated. Therefore, the maximum number of connected devices would increase by a factor of two.

### 2.1. m-CAP Modulator

**Figure 3:**
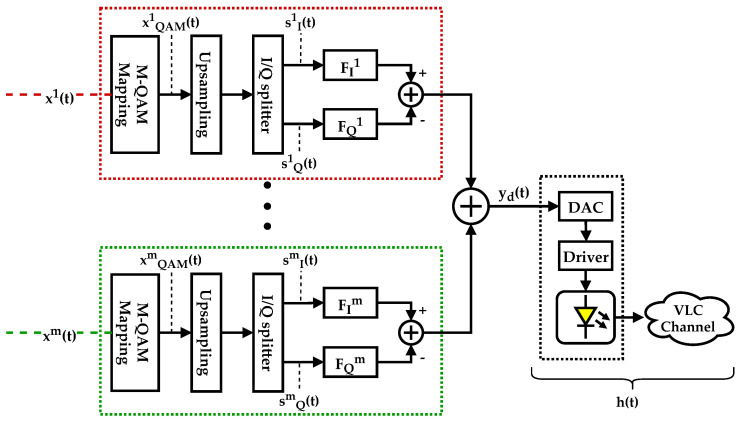
m-CAP modulator in the VLC base station.

Figure 3 presents the emitter’s block diagram, i.e., the digital m-CAP modulator. It represents several *m* data streams, intended for different users, each modulated in Quadrature Amplitude Modulation (QAM), formatted by two pass band filters (Hilbert pair) and added in the time domain to generate a digital m-CAP modulated signal, $y_{d}\left(t\right)$. This signal is then converted to the analog domain and transmitted as light with an LED. The impulse response h(t) represents the combined impulse responses of the Digital-to-Analog Converter (DAC), the LED driver circuit, the LED, and the VLC channel.

The following analysis establishes the mathematical model of the m-CAP digital modulator. Considering *m* as the index of independent data sources, the $m^{th}$ data signal is denoted by $x^{m}\left(t\right)$, where *t* is the time. Prior to QAM modulation, the given QAM symbols are:

(1) $$x_{QAM}^{m}\left(t\right)=A^{m}\left(t\right)+jB^{m}\left(t\right)$$

where $A^{m}$ and $B^{m}$ are the in-phase and quadrature components for the $m^{th}$ data source, respectively. The QAM symbols are then upsampled by a factor of *L* by means of zero padding. The upsampled signals are denoted by $s_{I}^{m}\left(t\right)$ and $s_{Q}^{m}\left(t\right)$. The upsampled QAM symbols are filtered by digital filters, $F_{I}^{m}\left(t\right)$ and $F_{Q}^{m}\left(t\right)$, the impulse responses of which form a Hilbert pair, given by, respectively:

(2) $$F_{I}^{m}\left(t\right)=p\left(t\right)cos\left(\omega_{c}^{m}t\right)$$

(3) $$F_{Q}^{m}\left(t\right)=p\left(t\right)sin\left(\omega_{c}^{m}t\right)$$

where p(t) is a pulse shaping filter and $\omega_{c}^{m}=2\pi f_{c}^{m}$, where $f_{c}^{m}$ is the central frequency on the $m^{th}$ band. Equation (4) gives the values for $f_{c}^{m}$:

(4) $$f_{c}^{m}=\frac{B}{2}\left(2m-1\right)$$

where *B* is the bandwidth of each band. The pulse shaping filter design will be addressed in Section 2.3.2.

The filter outputs are added at the modulator output, resulting in the following digital signal:

(5) $$y_{d}\left(t\right)=\sqrt{2}\sum_{m=1}^{M}\left(s_{I}^{m}\left(t\right)*F_{I}^{m}\left(t\right)-s_{Q}^{m}\left(t\right)*F_{Q}^{m}\left(t\right)\right)$$

Note that, if only the odd bands are modulated, the $s_{I}^{m}\left(t\right)$ and $s_{Q}^{m}\left(t\right)$ contribution is zero for m=2i−1,∀i∈N.

In order to transmit the $y_{d}\left(t\right)$ signal, the LED current must be modulated by an analog signal. This means that the digital voltage signal must be converted to an analog current, using a DAC and LED driver, which do not have unitary impulse responses. The emitter optical frontend usually has a low-pass filter characteristic due to the ADC anti-imaging filter, the LED driver, and the LED itself. DACs require a low-pass frequency response to remove the image bands resulting from the sampling process, which by the Nyquist sampling theorem must have its cut-off frequency less than half of the sampling frequency. Regarding LED drivers, parasitics in the driver components result in bandwidth limitations for high frequencies. Typical high-brightness white LEDs used in lighting systems have a cut-off frequency of a few MHz due to the phosphor coating [46]. Finally, the indoor VLC channel has an impulse response, the contributions of which usually arise from signal attenuation and signal reflections, resulting in Signal-to-Noise Ratio (SNR) degradation and interference phenomena such as intersymbol interference [47,48]. Note that the VLC channel often presents a non-flat frequency response, which may degrade the SNR, in particular for the higher frequency bands [49]. The combined impulse responses of the described process will be denominated as h(t). However, due to the narrowband characteristic of each carrier in the proposed architecture, h(t) was considered to have a low-pass response with a constant attenuation in the pass band of each user, i.e., the frequency response is considered to be flat for a particular band.

### 2.2. Analog CAP Demodulator

**Figure 4:**
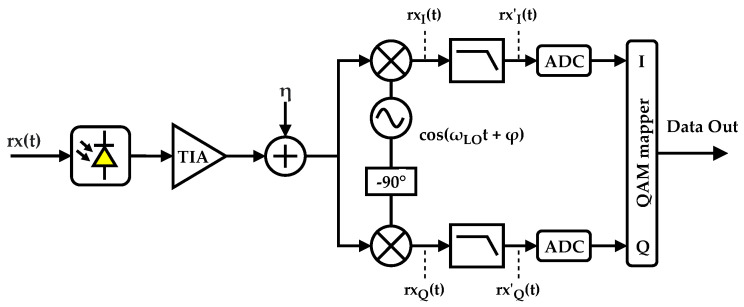
Analog CAP receiver. TIA, Transimpedance Amplifier.

At the receiver side, independent devices need to demodulate a single band bearing in mind the IoT requirements of low cost and low power consumption. The receiver diagram is shown in Figure 4: the optical signal is translated to a current by a photodiode having a responsivity of *ℜ*, and a transimpedance gain, *A*, is applied. A noise source, n(t), is added in order to include the system noise. The signal is then multiplied in each branch by a sinusoidal wave, with a phase shift of 90${}^{\circ }$ between them, with angular frequency $\omega_{LO}$, plus a constant phase, ϕ. The mixing stage shifts the received signal to its baseband form. Following the mixing stage, a filtering stage with low-pass filters is used to remove both the undesired and imaging bands. The impulse response of the filters is denoted by g(t). Analog-to-digital conversion occurs to sample the demodulated symbols at the rate *R*, which after a QAM de-mapping process, results, ideally, in the original data stream.

When compared to its digital equivalent, the analog demodulator uses less active components, thus requiring less power and reducing its overall cost. A digital CAP demodulator would require complex Digital Signal Processors (DSPs), demanding hundreds of logical gates implemented by thousands of transistors. Instead, the analog demodulator requires a Local Oscillator (LO), two mixers (implemented with few transistors), and analog filtering stages, severely reducing its cost and power consumption. Another advantage of using an analog frontend is the required sample rate of the receiver ADCs, which are much slower when compared with the digital demodulator. In other words, in digital demodulation, the signal must be sampled according to the Nyquist sampling theorem, i.e., more than twice the signal bandwidth (of all bands). Instead, using the analog frontend, the ADCs’ sampling frequency can be lowered to the symbol rate. Therefore, the sampling frequency can be decreased by a factor proportional to the number of bands.

At the input of the quadrature mixer, the signal is given by:

(6) $$rx\left(t\right)=ℜA\left[y_{d}\left(t\right)*h_{t}\left(t\right))\right]+\eta \left(t\right)$$

where η(t) is considered to be Additive White Gaussian Noise (AWGN) resulting from the photodiode shot noise and the Transimpedance Amplifier (TIA) noise [50].

The mixers multiply the rx(t) signal by a sinusoidal wave, with the central frequency of the desired band, $\omega_{LO}$, having a 90${}^{\circ }$ phase shift between each branch. Assuming ϕ=0, i.e., the LO is in phase with rx(t), the mixers’ signal output is given by,

(7) $$rx_{I}\left(t\right)=rx\left(t\right)cos\left(\omega_{LO}t\right)$$

(8) $$rx_{Q}\left(t\right)=rx\left(t\right)cos\left(\omega_{LO}t-\frac{\pi }{2}\right)=-rx\left(t\right)sin\left(\omega_{LO}t\right)$$

In order to simplify the mathematical analysis of the demodulation, $h_{t}\left(t\right)$ will be assumed unity in the forgoing analysis. By combining Equations (6)–(8), as well as the usage of the convolution definition in the Equation (5) convolution operations, the in-phase and quadrature signals are demodulated as follows:

(9) $$rx_{I}\left(t\right)=\left[ℜA\sqrt{2}\sum_{m=1}^{M}\int_{-\infty }^{\infty }\left(s_{I}^{m}\left(\tau \right)F_{I}^{m}\left(t-\tau \right)-s_{Q}^{m}\left(\tau \right)F_{Q}^{m}\left(t-\tau \right)\right)d\tau +\eta \left(t\right)\right]cos\left(\omega_{LO}t\right)$$

(10) $$rx_{Q}\left(t\right)=-\left[ℜA\sqrt{2}\sum_{m=1}^{M}\int_{-\infty }^{\infty }\left(s_{I}^{m}\left(\tau \right)F_{I}^{m}\left(t-\tau \right)-s_{Q}^{m}\left(\tau \right)F_{Q}^{m}\left(t-\tau \right)\right)d\tau +\eta \left(t\right)\right]sin\left(\omega_{LO}t\right)$$

By replacing $F_{I}$ and $F_{Q}$, we obtain:

(11) $$\begin{array}{c} rx_{I}\left(t\right)=[ℜA\sqrt{2}\sum_{m=1}^{M}\int_{-\infty }^{\infty }(s_{I}^{m}\left(\tau \right)p\left(t-\tau \right)cos\left(\omega_{c}^{m}\left(t-\tau \right)\right)-\\ \;\;\;\;\;\;\;\;\;\;-s_{Q}^{m}\left(\tau \right)p\left(t-\tau \right)sin\left(\omega_{c}^{m}\left(t-\tau \right)\right))d\tau +\eta \left(t\right)]cos\left(\omega_{LO}t\right) \end{array}$$

(12) $$\begin{array}{c} rx_{Q}\left(t\right)=-[ℜA\sqrt{2}\sum_{m=1}^{M}\int_{-\infty }^{\infty }(s_{I}^{m}\left(\tau \right)p\left(t-\tau \right)cos\left(\omega_{c}^{m}\left(t-\tau \right)\right)-\\ \;\;\;\;\;\;\;\;\;\;-s_{Q}^{m}\left(\tau \right)p\left(t-\tau \right)sin\left(\omega_{c}^{m}\left(t-\tau \right)\right))d\tau +\eta \left(t\right)]sin\left(\omega_{LO}t\right) \end{array}$$

Considering phase alignment between $cos\left(\omega_{c}^{m}\left(t-\tau \right)\right)$ and $cos(\omega_{LO}t)$, meaning that $\tau \;=\;2n\pi ,\forall n\in N_{0}$, trigonometric products arise from the equations:

(13) $$\begin{array}{c} rx_{I}\left(t\right)=\frac{ℜA\sqrt{2}}{2}\sum_{m=1}^{M}\int_{-\infty }^{\infty }\;(s_{I}^{m}\left(\tau \right)p\left(t-\tau \right)\left[cos\left((\omega_{c}^{m}-\omega_{LO})\tau \right)+cos\left((\omega_{c}^{m}+\omega_{LO})\tau \right)\right]-\\ \;\;\;\;\;\;\;\;\;\;-s_{Q}^{m}\left(\tau \right)p\left(t-\tau \right)\left[sin\left((\omega_{c}^{m}-\omega_{LO})\tau \right)+sin\left((\omega_{c}^{m}+\omega_{LO})\tau \right)\right])d\tau +\eta \left(t\right)cos\left(\omega_{LO}t\right) \end{array}$$

(14) $$\begin{array}{c} rx_{Q}\left(t\right)=-\frac{ℜA\sqrt{2}}{2}\sum_{m=1}^{M}\int_{-\infty }^{\infty }\;(s_{I}^{m}\left(t\right)p\left(t-i\tau \right)\left[sin\left((\omega_{c}^{m}+\omega_{LO})\tau \right)-sin\left((\omega_{c}^{m}-\omega_{LO})\tau \right)\right]-\\ \;\;\;\;\;\;\;\;\;\;-s_{Q}^{m}\left(t\right)p\left(t-\tau \right)cos\left((\omega_{c}^{m}-\omega_{LO})\tau \right)-cos\left(\left(\omega_{c}^{m}+\omega_{LO}\right)\tau )\right])d\tau -\eta \left(t\right)sin\left(\omega_{LO}t\right) \end{array}$$

Equations (13) and (14) are the mathematical description of the signal at the receiver mixers’ output. High-frequency signal components are discarded by the usage of low-pass filters at the mixers’ output, g(t), with a cut-off frequency around $\omega_{c}^{1}$. Considering f(t)=p(t)∗g(t), the signals at the filter output are given by:

(15) $$\begin{array}{c} rx_{I}^{\prime }\left(t\right)=\frac{ℜA\sqrt{2}}{2}\sum_{m=1}^{M}\int_{-\infty }^{\infty }(s_{I}^{m}\left(\tau \right)f\left(t-\tau \right)cos\left((\omega_{c}^{m}-\omega_{LO})\tau \right)-\\ \;\;\;\;\;\;\;\;\;\;-s_{Q}^{m}\left(\tau \right)f\left(t-\tau \right)sin\left((\omega_{c}^{m}-\omega_{LO})\tau \right))d\tau +\eta \left(t\right)cos\left(\omega_{LO}t\right)*g\left(t\right) \end{array}$$

(16) $$\begin{array}{c} rx_{Q}^{\prime }\left(t\right)=-\frac{ℜA\sqrt{2}}{2}\sum_{m=1}^{M}\int_{-\infty }^{\infty }(-s_{I}^{m}\left(\tau \right)f\left(t-\tau \right)sin\left((\omega_{c}^{m}-\omega_{LO})\tau \right)-\\ \;\;\;\;\;\;\;\;\;\;-s_{Q}^{m}\left(\tau \right)f\left(t-\tau \right)cos\left((\omega_{c}^{m}-\omega_{LO})\tau \right))d\tau -\eta \left(t\right)sin\left(\omega_{LO}t\right)*g\left(t\right) \end{array}$$

If $\omega_{LO}=\omega_{c}^{m}$, some of the trigonometric terms cancel out, and by applying the convolution definition, the output signals are:

(17) $$rx_{I}^{\prime }\left(t\right)=\frac{ℜA\sqrt{2}}{2}\left(s_{I}^{m}\left(t\right)*p\left(t\right)*g\left(t\right)\right)+\eta \left(t\right)cos\left(\omega_{LO}t\right)*g\left(t\right)$$

(18) $$rx_{Q}^{\prime }\left(t\right)=\frac{ℜA\sqrt{2}}{2}\left(s_{Q}^{m}\left(t\right)*p\left(t\right)*g\left(t\right)\right)-\eta \left(t\right)sin\left(\omega_{LO}t\right)*g\left(t\right)$$

Equations (17) and (18) show that, by carefully selecting a pair of filters p(t) and g(t), it is possible to recover the original symbols $s_{I}^{m}\left(t\right)$ and $s_{Q}^{m}\left(t\right)$. Nevertheless, the received symbols may have a non-unitary gain, and bandwidth limited noise is added.

### 2.3. Filters

In order to recover the transmitted symbols, $s_{I}\left(t\right)$ and $s_{Q}\left(t\right)$, with improved SNR, the receiver filter should be a matched filter for p(t) [51]. However, there is no implementation of an analog matched filter for a raised cosine filter since its impulse response is non-causal. Nevertheless, the recovery of the symbols can be achieved by applying a filter that has approximate characteristics to the raised cosine filter. Thus, the filter stage should be properly designed in order to maximize the system performance. Since the receiver filter has more implementation constraints, compared to the digital transmission filter, it will be first analyzed in the following subsections.

#### 2.3.1. Receiver Filter

There are several requirements that should be taken into account when designing the receiving filters: (i) filter order: higher performance for higher orders, but difficult to implement; (ii) filter cut-off frequency: if closer to the band limit, it could attenuate the desired signal; (iii) frequency response: it should attenuate the undesirable bands, including image bands from the mixing process, while having low and flat attenuation in the pass band; (iv) impulse response: the output signal should not be severely distorted under the risk of receiving errors. This last aspect may be determinant of a successful demodulation, since the filters need to remove high-frequency components while maintaining the baseband signal integrity. Hence, p(t) and g(t) need to be selected to have a constant group delay in the baseband bandwidth, i.e., the filter pass band. This property ensures the digital data, which are PAM pulses of the in-phase and quadrature components of the QAM symbols, do not suffer different delays for different frequencies. Note that the frequency spectrum of a PAM signal is a sincwaveform, which has an infinite bandwidth; however, most of the energy is concentrated in the central lobe [51]. Therefore, considering the absence of a receiver equalizer in IoT devices (for the sake of low complexity and cost), one of the main requirements for the receiving filter is a constant group delay.

**Figure 5:**
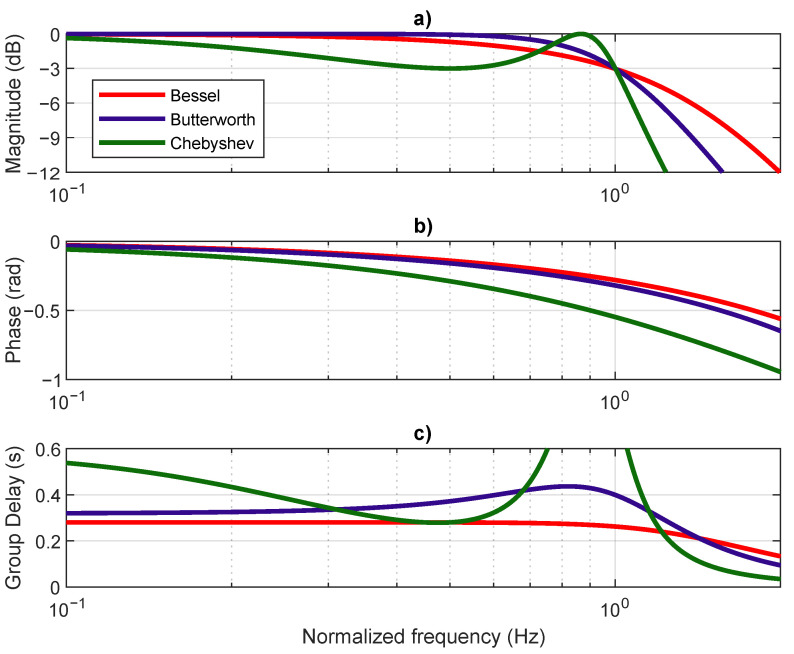
Analog third-order filter types’ comparison: (**a**) magnitude, (**b**) phase, and (**c**) group delay.

Two filter types were considered for the receiver filter, g(t): (i) Bessel and (ii) Butterworth. Bessel filters are known for having a flat group delay and are commonly used in digital communication systems as a transmission filter. Nevertheless, their frequency response performance is poor when compared to other filter types such as Butterworth or Chebyshev filters, particularly concerning the attenuation decay after the cut-off frequency. Figure 5a presents the normalized filters’ frequency response, when all are designed to have a magnitude of −3 dB at the cut-off frequency of 1 Hz, Figure 5b,c shows the phase and the group delay for the Bessel, Butterworth, and Chebyshev filter types, with respect to the normalized frequency. It is observable that Bessel has the slowest decay, while Chebyshev has the fastest (although with a ripple in the pass band). Regarding the group delay, the Bessel filter has the flattest pass band response, while Chebyshev has a great amount of variation. The Butterworth filter presents a compromise between Bessel and Chebyshev, valid for both magnitude and group delay response. Due to its wide group delay variations, Chebyshev filters were not considered in further analysis, for the proposed system.

**Figure 6:**
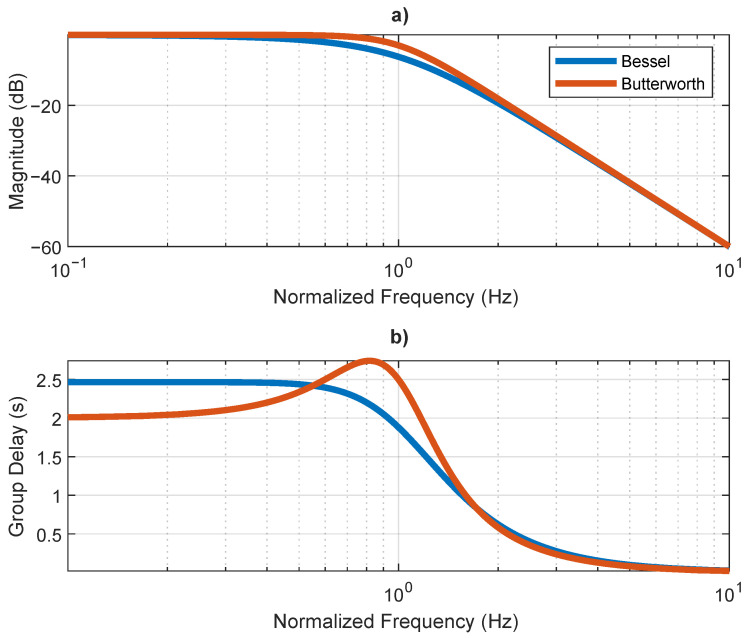
Comparison between Bessel and Butterworth third order filters, considering high-frequency asymptote normalization: (**a**) magnitude and (**b**) group delay.

Unlike other filters, the Bessel filter −3 dB cut-off frequency is not constant with the filter order, leading to an attenuation higher than 3 dB at the desired cut-off frequency. The desire cut-off frequency can be determined asymptomatically by analyzing the high-frequency behavior [52]. In contrast with Figure 5, Figure 6 shows a Bessel filter where a high-frequency response is asymptotically coincident with the response of a Butterworth filter, both order three. This operation is achieved by normalizing the filter poles and gain, as described in [52]. An asymptotic analysis of the high-frequency response of the Bessel filter reveals a normalized cut-off frequency of 1 Hz, equal to the Butterworth filter. However, the magnitude at 1 Hz is −3 dB and −6.24 dB for the Butterworth and Bessel filters, respectively. The attenuation suffered by the Bessel filters in the asymptotic cut-off frequency increases as the filter order increases, which results in an increasingly non-flat amplitude response in the pass band, with increasing filter order. Both filters present non-flat group delay. Nevertheless, the Butterworth filter presents non-flat group delay at lower frequencies.

#### 2.3.2. Emitter Filter

Contrary to the receiver, the emitter has higher flexibility concerning the filter design, since it allows digitally setting the filter impulse response. The digital filter is designed according to Equations (2) and (3), with particular attention to the pulse shaping filter, p(t). This section describes the two considered approaches to design p(t): (a) the raised cosine filter and (b) the filter matched to the analog receiver filter (designated here as the matched filter approach).

(a) Raised cosine filters: 

A common pulse shaping filter used in digital communication systems is the raised cosine filter, which is able to mitigate Intersymbol Interference (ISI) in communication systems. Its coefficients are computed as follows:

(19) $$p\left(t\right)=\frac{sin(\frac{\pi t}{T})}{\frac{\pi t}{T}}\frac{cos(\frac{\pi \beta t}{T})}{1-{\left(\frac{2\beta t}{T}\right)}^{2}}$$

where T=1/R is the data symbol period and β∈[0,1] is the filter roll-off factor. The filter bandwidth is given by:

(20) $$B^{\prime }=\frac{R}{2}\left(\beta +1\right)$$

**Figure 7:**
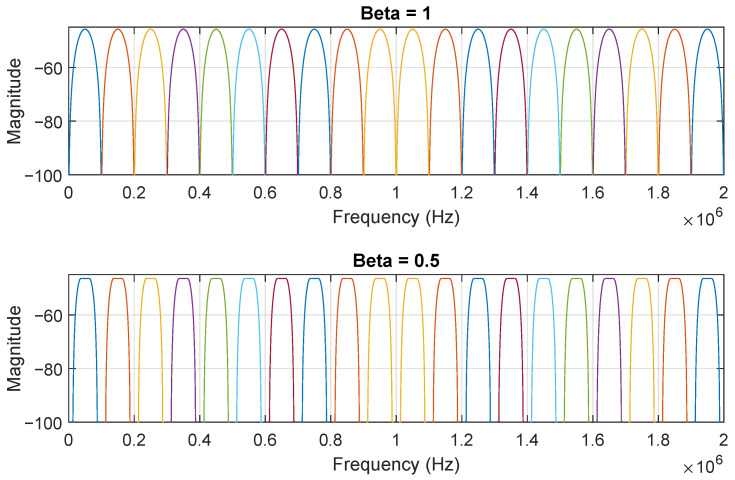
m-CAP frequency spectrum for β=1 and β=0.5: as β decreases, the band spacing increases.

From Equation (20), using a lower β value, a higher data rate, *R*, can be transmitted using the same bandwidth, $B^{\prime }$, up to two-times higher, improving spectral efficiency. From the literature, many authors studied the impact of changing β, trying to optimize the systems for a high data rate by increasing *R* for the same $B^{\prime }$. However, a high data rate is not a priority in most of the IoT systems, in particular for the proposed system, the goal of which is to demodulate an m-CAP signal with low resources. Hence, fixing *R* while varying β allows a bandwidth compression: for the same *R*, as β decreases, $B^{\prime }$ also decreases. There are two main reasons to fix *R* while varying β:

- Bearing in mind the receiver architecture’s simplicity, the synchronism mechanism can be simplified if there is no constellation rotation between each symbol. CAP modulation is known to have a deterministic phase shift between each symbol [53]. Although the phase shift between modulated symbols is deterministic, the receiver would require a complex device synchronization mechanism to accommodate the signal periodic phase shift. In other words, using β≠1 would require the receiver to continuously adjust the LO phase for each received symbol. This can be avoided if R=B/2, since according to Equation (4), the $m^{th}$ carrier has a period that is a multiple of 1/R.
- Analog demodulation with a continuous spectrum may negatively impact the data recovery due to the non-ideal frequency response of analog filters, i.e., it has a finite value decay. Therefore, by fixing R=B/2 and varying β, guard bands are created as shown in Figure 7.

(b) Matched filters: 

As previously mentioned, a matched filter is commonly used to improve the SNR in communication systems [51]. A matched filter is the optimal linear filter that allows SNR maximization and is derived from the original filter as follows:

(21) g(t)=p(τ−t)

where g(t) is a matched filter for the p(t) filter and τ is a time delay. In a fully digital communication system, the matched filter is chosen for both the emitter and receiver bearing in mind the desired impulse response for the convolution of both filters. For instance, the square-root raised cosine filter is commonly used, both in the emitter and receiver. The convolution between both gives the raised cosine filter impulse response, resulting in an ISI-free communication [51]. However, as said, the raised cosine filter is not realizable in the analog domain. In the proposed system, the filter choice is limited by the receiver analog implementation; thus, a matched filter should be digitally designed in the emitter. Therefore, the emitter pulse shaping filter, p(t), should be generated from the impulse response of the receiver filter, g(t). Note that the peak of p(t) should be aligned with the peak of the cosine and sine terms of Equations (2) and (3), to avoid losses in the signal energy.

## 3. Simulation Setup

**Figure 8:**
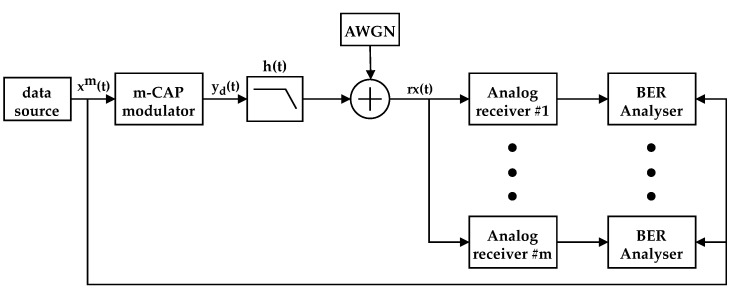
Simulation model block diagram.

The proposed system was simulated in MATLAB^®^ Simulink^®^. A block diagram of the simulation model is shown in Figure 8. The “data source” block generates the $m^{th}$ independent uniformly distributed pseudorandom symbols, $x^{m}\left(t\right)$, which are then modulated by the “m-CAP modulator”, resulting in the $m^{th}$ independent modulated bands, corresponding to the $y_{d}\left(t\right)$ signal. A low-pass filter is added at the modulator output, which corresponds to h(t), the combined impulse response of the DAC, LED driver, LED, and VLC channel. The VLC channel response is considered to be unitary. AWGN is added to the received signal, which is demodulated by the $m^{th}$ independent analog receiver. A BER analysis was performed at the output of each receiver, by comparing the demodulated data with the original data, for each band. The simulation ran until it reached one of two conditions: (1) 100 errors were found; (2) $10^{7}$ symbols were simulated. In order to guarantee good resolution for lower Eb/No, at least 2000 symbols were simulated for every point. Table 1 summarizes the general simulation parameters used in the simulation setup.

Two filter types were considered for the analog receiver: Bessel and Butterworth, both of the third order. Regarding the emitter, three pulse shaping filters, p(t), were studied: raised cosine filter, matched Bessel filter, and matched Butterworth filter. Table 2 shows the parameters chosen for the raised cosine filter.

The implementation of $F_{I}^{m}$ and $F_{Q}^{m}$ followed a Finite Impulse Response (FIR) filter architecture. In order to design the raised cosine filter, a symbol span of 10 was selected in order to effectively mitigate the energy in the side lobes, due to the filter time span truncation. The samples per symbol, SPS, parameter is defined as:

(22) $$SPS=\left\lceil \frac{F_{s}}{R}\right\rceil$$

**Figure 9:**
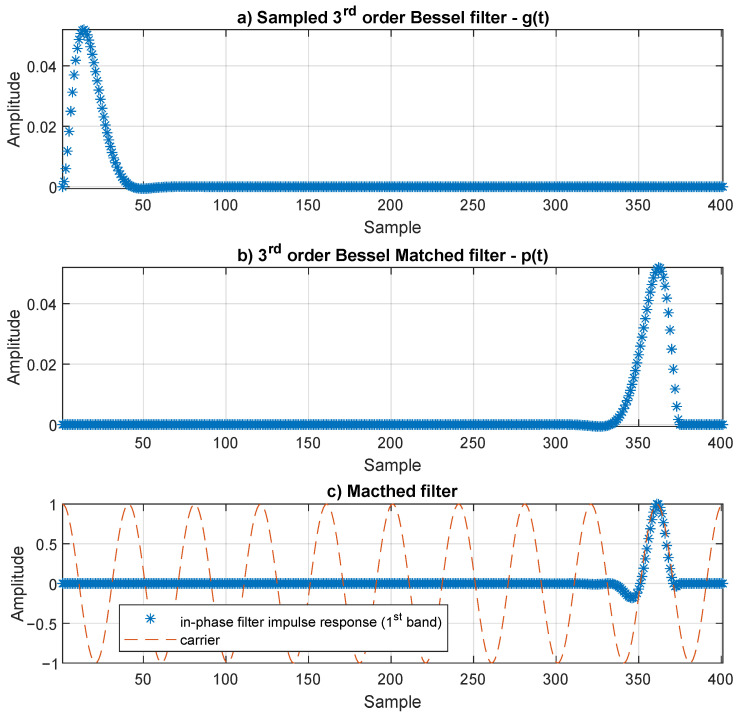
Matched filter generation: (**a**) impulse response of a third-order Bessel filter, g(t), sampled at $F_{s}$; (**b**) matched filter, p(t), consisting of the flipped impulse response of (**a**), with zero padding at the end; (**c**) normalized impulse response of the in-phase filter and the correspondent carrier (first band).

From Equation (22), the SPS parameter assumes a value of 40. The total number of coefficients of the designed raised cosine filter is 401; hence, $F_{I}^{m}$ and $F_{Q}^{m}$ will also have the same number of coefficients. In order to allow a fair comparison between the raised cosine filters and the matched filters, the same number of coefficients for all the simulation scenarios was considered, regardless of the p(t) used. The Figure 9 illustrates the matched filter generation steps.

The generation of the matched filters considered the original analog filters’ impulse response. The impulse response of the analog filter, g(t), was sampled at $F_{s}$, and the obtained samples (Figure 9a) would directly be the coefficients of an FIR filter, which were time reversed. Moreover, in order to guarantee synchronism between the matched filter impulse response and the carrier, a negative delay was introduced: by doing this, the filter coefficients were shifted to the left side. The remaining coefficients, at the end, were set to zero (Figure 9b). Considering that g(t) is a third-order Bessel filter, the matched filter, p(t), generation is shown in Figure 9, as well as the first band in-phase filter impulse response of the m-CAP modulator (Figure 9c).

## 4. Results

This section presents the results obtained by the simulations. Table 3 summarize the performed simulations, highlighting the main parameters for each case. The simulations were divided into three main groups: (i) a single modulated band, (ii) all 10 bands being modulated, and (iii) only the odd bands being modulated (as shown in Figure 2). For each group, two Rx filters, g(t), were evaluated: Bessel and Butterworth. In each case, two Tx filters were evaluated: (i) a raised cosine filter, including different β values, and (ii) a matched filter.

**Figure 10:**
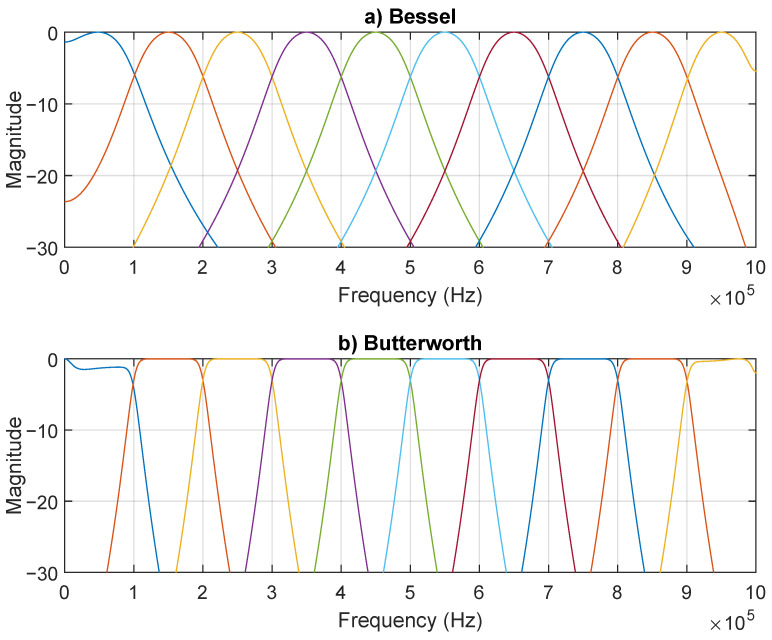
Normalized frequency spectrum considering a matched filter (third order): (**a**) Bessel and (**b**) Butterworth.

Previously, Figure 7 presented the frequency spectrum for $y_{d}\left(t\right)$ when p(t) was a raised cosine filter. Figure 10 shows the output signal normalized frequency spectrum, however using the generated matched Bessel and matched Butterworth filters as p(t). As seen, the energy was more spread in the case of the Bessel when compared with the Butterworth.

**Figure 11:**
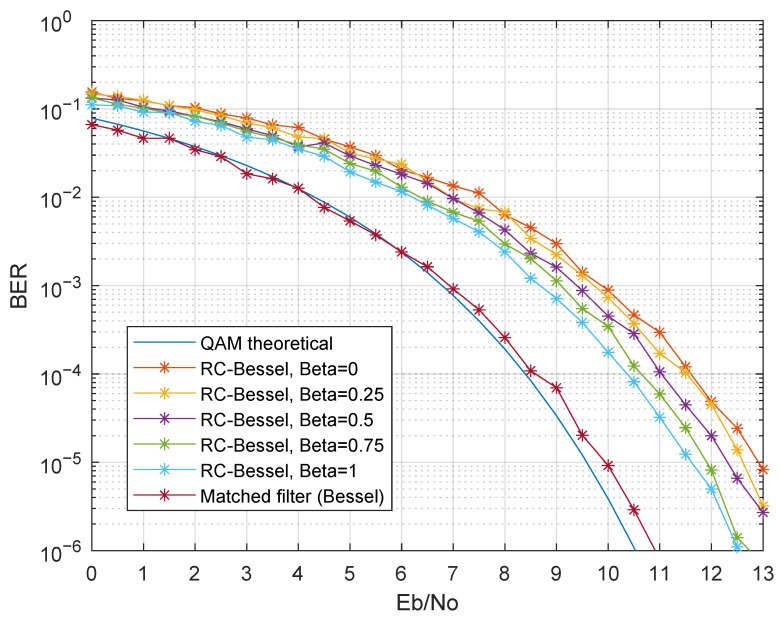
BER performance comparison for a single modulated band-Rx filter: third-order Bessel; p(t): raised cosine filter (changing β) and the matched Bessel filter (Simulations 1 and 2).

The first presented results evaluated the performance with only one modulated band, in particular the first band. Therefore, inter-band interference was not present in these results. Figure 11 presents the simulation results considering g(t) as a third-order Bessel filter, for various p(t) (Simulation Numbers 1 and 2 of Table 3). Regarding the raised cosine filter, there was a BER degradation as β decreased. This was mainly due to the raised cosine impulse response change as β changed: lower roll-off factors had a higher ripple in the impulse response, thus resulting in higher ISI in non-ideal scenarios, in particular when g(t) was not a matched filter and did not guarantee ISI-free communication. The matched filter in the receiver presented an improved BER when compared to the raised cosine filter, as expected. The simulated BER curve of the matched filter was almost coincident with the theoretical QAM BER curve for the same Eb/No, showing a remarkable improvement when compared with the RC-Bessel setup.

**Figure 12:**
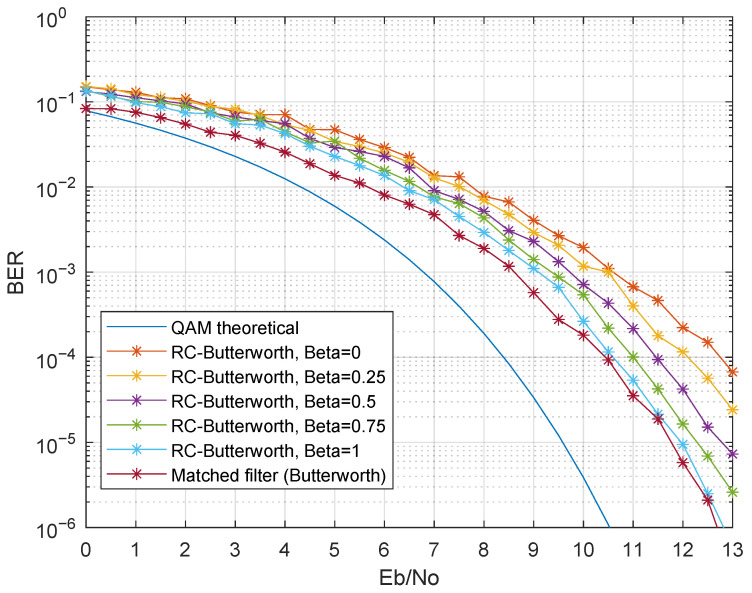
BER performance comparison for a single modulated band-Rx filter: third-order Butterworth; p(t): raised cosine filter (changing β) and the matched Butterworth filter (Simulations 3 and 4).

The same scenarios were simulated for the Butterworth filter as g(t), which are presented in Figure 12 (Simulation Numbers 3 and 4). Identical performance was observed for the raised cosine filter as p(t), when compared with Figure 11’s results. However, there was an Eb/Nodegradation, in particular for lower β values. Regarding the matched Butterworth filter as p(t), it presented a better performance than the raised cosine filter. Nevertheless, the matched Bessel filter showed the best performance for scenarios without inter-band interference. Since every parameter was identical, except for the filter type, it can be concluded that the group delay characteristics of Bessel filters were essential for improving the demodulation performance.

**Figure 13:**
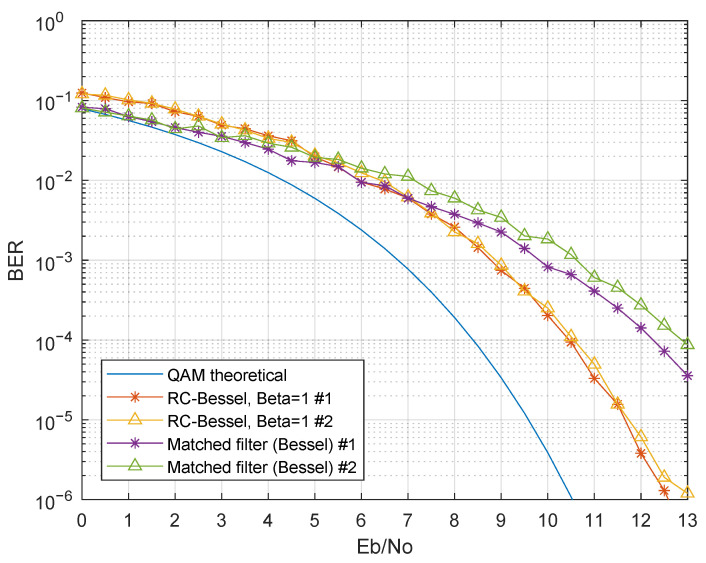
BER performance comparison with inter-band interference-Rx filter:third-order Bessel; p(t): raised cosine filter (β=1) and the matched Bessel filter (Simulations 5 and 6).

The next results show the system performance in a more realistic scenario, where multiple bands may be simultaneously modulated. The considered simulation parameters were identical to the single-band scenarios. However, all the bands were modulated, and the BER was evaluated at the first and second bands. Figure 13 shows the BER performance for the first two bands (# 1 and # 2), with a third-order Bessel filter as g(t), referring to Simulations 5 and 6. For better visualization purposes, the raised cosine roll-off factor was only evaluated for β=1, since it presented the best performance in the single-modulated band results.

**Figure 14:**
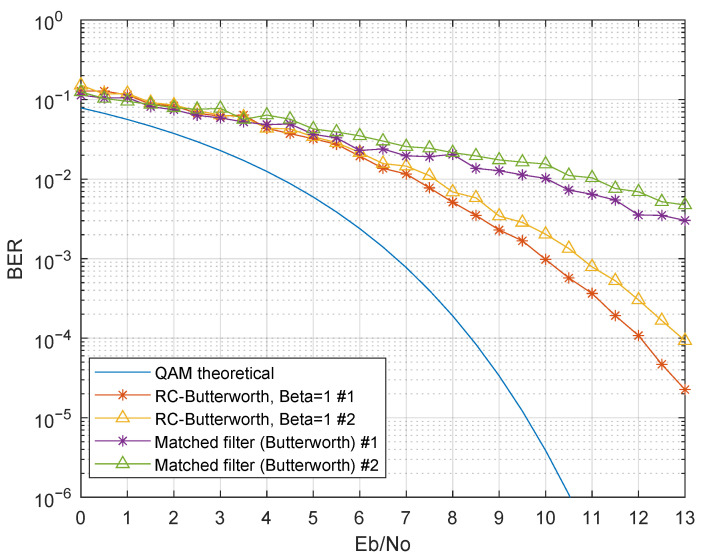
BER performance comparison with inter-band interference-Rx filter:third-order Butterworth; p(t): raised cosine filter (β=1) and the matched Butterworth filter (Simulations 7 and 8).

Comparing the performance with and without inter-band interference, there was a performance degradation when all the bands were being modulated. Although using a raised cosine filter as p(t) presented almost the same performance as before, the BER significantly degraded when the raised cosine was replaced by a matched Bessel filter. This was obviously due to the non-bandwidth limited characteristic of Bessel filters, as opposed to the raised cosine filters, which resulted in high inter-band interference. The same behavior occurred when using a matched Butterworth filter as g(t), as shown in Figure 14 (Simulations 7 and 8), however showing even worse BER performance using the matched filter. The previous results show that the Bessel filters achieved better performance when compared with Butterworth filters. Nevertheless, performance was severely degraded in scenarios where inter-band interference occurred.

**Figure 15:**
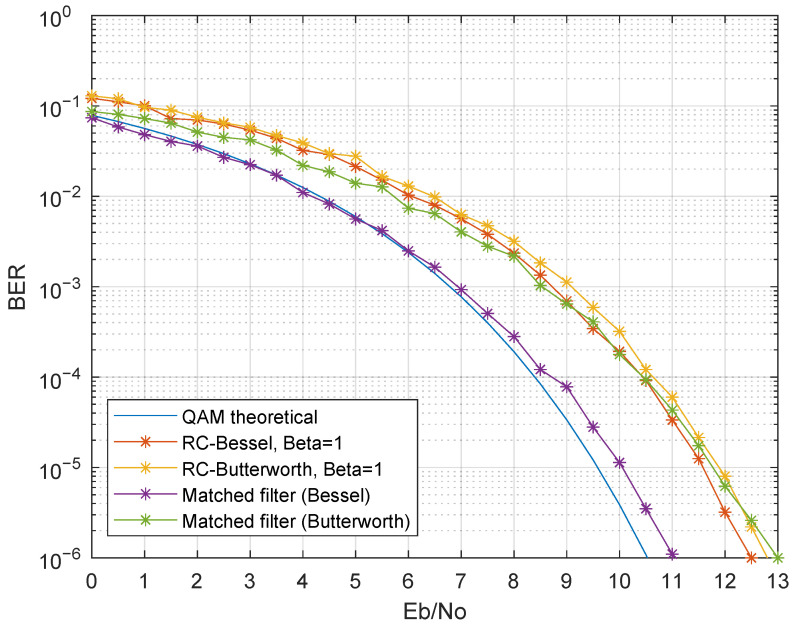
BER performance using guard bands, by suppressing the even bands (Simulations 9 to 12).

The results in Figure 15 consider the suppression of the even bands (Simulations 9 to 12). For visualization purposes, only β=1 is shown for the raised cosine filter, and only the BER in the first band BER is shown. The BER curves show identical results when compared to Simulations 1 to 4, thus effectively mitigating the inter-band interference and with a remarkable performance when matched Bessel filters were considered.

Although this guard band scheme degraded the spectral efficiency in half, the simplification in the receiver design can be easily achieved, allowing the desired IoT requirements for low-cost, low-power, and small-sized devices.

**Figure 16:**
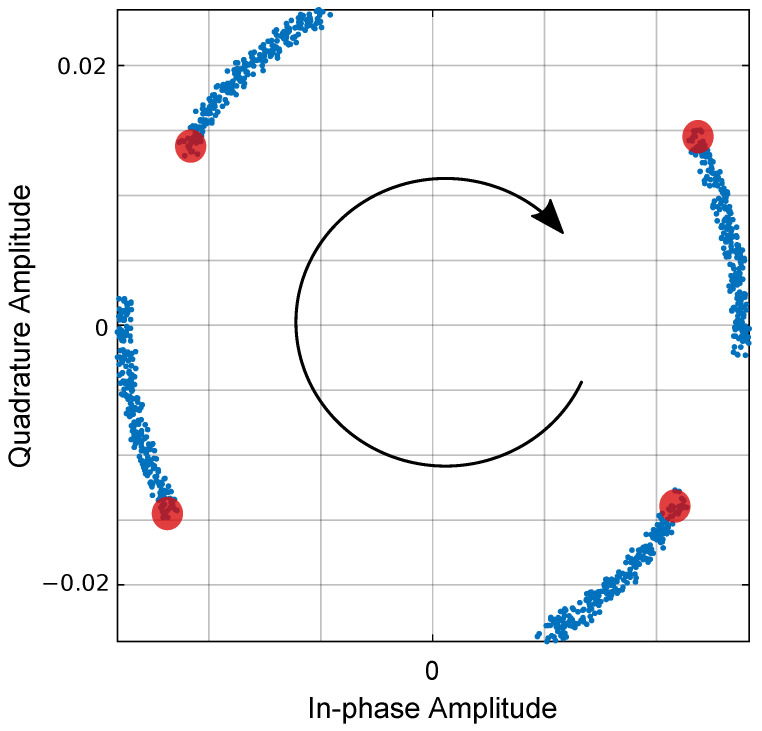
Received QAM constellation for the first 1000 symbols in the first band, showing constellation rotation in the presence of a frequency shift in the receiver Local Oscillator (LO), of 5 Hz @ $f_{c}^{1}$ = 50 kHz: red circles, received symbols without frequency shift; blue points, received symbols.

**Figure 17:**
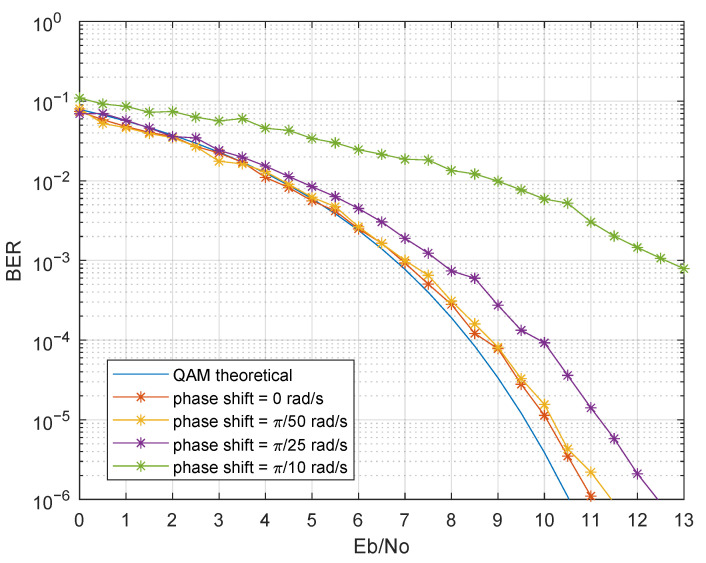
BER performance considering constant phase shift in the receiver LO for {0,π/50,π/25,π/10} rad/s.

The proposed system uses a local oscillator that may present non-ideal parameters, namely: (i) frequency shift, (ii) phase shift, and (iii) phase noise. The following analysis considered the non-ideal parameters in synchronization in the scenario of Simulation 10. Regarding frequency shift, the system was highly sensitive if the frequencies of the local oscillator, $\omega_{LO}$, and the emitter filter sine wave, $\omega_{c}^{m}$, did not match. Exact oscillator frequency is not feasible in real implementations due to component tolerances and thermal effects. From Equations (15) and (16), the sine and cosine terms only canceled out if both frequencies were exactly the same. In the case of a frequency shift at one of the sides, a cumulative phase delay was added; hence, the received constellation was continuously rotating (Figure 16). Without the presence of a proper carrier recover scheme in the receiver, the symbols’ demodulation can not be correctly performed. Furthermore, phase shift, also known as phase offset, may arise in real implementations, resulting in a degradation of BER as the phase shift increases, as shown in Figure 17.

**Figure 18:**
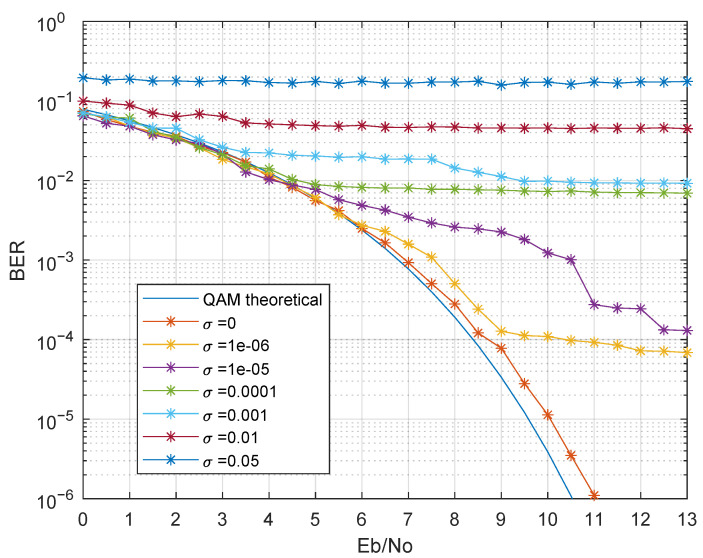
BER performance considering phase noise.

Both problems can be solved with system synchronization, which can be accomplished using the carrier frequency recovered from the suppressed carrier modulation, using for instance a Costas loop [54]. The synchronization will often actuate in a Voltage Controlled Oscillator (VCO) in a feedback loop, in order to match the LO frequency and phase with the carrier in the received signal. Hence, the resulting LO frequency will depend on the synchronism loop characteristic, resulting in voltage error and noise in the voltage at the VCO input, considered to be AWGN with zero mean and variance σ. The noise at the VCO input will cause a fast switch in VCO synthesized frequency, introducing phase noise in the demodulation. For simulation purposes, a VCO with a quiescent frequency $f_{c}$ Hz and a sensitivity of $f_{c}$ Hz/V was considered. Figure 18 presents the BER results of the considered scenario, varying the σ of the input voltage AWGN.

As observed in Figure 18, even small values of σ led to a degradation in the BER, imposing a lower bound. Note that the small values of σ need to be analyzed taking into account the high VCO sensitivity, which normalizes the value of σ. Therefore, considering σ = 10${}^{-6}$ and $f_{c}$ = 50 kHz, about 68.3% of the frequency values had a frequency error lower than 0.05 Hz. In this scenario, the BER performance was severely degraded for Eb/No < 9 dB.

## 5. Conclusions

This paper proposed a VLC-based IoT system architecture, where the emitter was implemented in the digital domain while the demodulation in the receiver was accomplished in the analog domain. Two system parameters were evaluated: the filtering stage and the usage of guard bands.

Concerning the filtering stage, two scenarios were considered: with and without inter-band interference. When a single band was modulated, i.e., no inter-band interference, it was shown that, using a raised cosine filter as the emitter pulse shaping filter with a roll-off factor of one and a Bessel or Butterworth filter in the analog receivers, the performance was degraded about 2 dB, when compared to the theoretical QAM BER performance in the presence of AWGN. The performance was even worse if the roll-off factor decreased due to the increase of the ISI, created by the filters’ mismatch. By using a matched filter in the digital emitter, the system performance was improved, in particular for Bessel filters, achieving a remarkable BER performance, with a 0.5 dB Eb/No gain loss when compared with the theoretical QAM modulation BER curve in the presence of AWGN. The improved performance obtained by the matched Bessel filter was due to its constant group delay characteristic, as opposed to Butterworth filters. However, when all the bands were modulated, inter-band interference arose, thus degrading the SNR. Although the results pointed out an overall performance decrease, the matched filters presented the worst results, in particular the Butterworth filters. In scenarios where contiguous bands were modulated, the best result was achieved when using a raised cosine filter with β=1 as p(t) and an analog Bessel filter.

Since high spectral efficiency is not a priority in VLC-based IoT systems, the usage of guard bands was considered in this study. The results stated that, by suppressing the even bands, the BER performance was identical to the scenarios where only one band was modulated, for all p(t) and g(t) configurations. Therefore, as previously, the best performance was achieved by using a digital matched Bessel filter as p(t) together with an analog Bessel filter. The usage of guard bands proved to be effective to mitigate inter-band interference.

In conclusion, the results showed that a VLC-based IoT system using a digital m-CAP modulator and analog receivers was feasible, achieving a BER identical to the fully digital implementation. In order to improve the system BER, guard bands were required, improving inter-band interference rejection, as well as a synchronism loop in the analog receivers. In particular, using a third-order analog Bessel filter, along with a quadrature mixer properly synchronized, the proposed architecture could achieve a BER lower than $10^{-3}$ for an Eb/No of 6 dB.

## Figures and Tables

**Table 1:** System simulation parameters.

Parameter	Value
Simulation time step	50 ps
Modulator sampling frequency ($F_{s}$)	2 MHz
LPFbandwidth (6th order) (h(t))	1 MHz
Number of bands (m)	10
Band bandwidth (B)	100 kHz
QAM modulation order	4
Symbol rate (*R*)	50 kS/s
Maximum simulated symbols	$10^{7}$
Minimum simulated symbols	2000
Number of error threshold	200

**Table 2:** Raised cosine filter simulation parameters.

RCFilter Parameter	Value
roll-off factor (β)	[0, 0.25, 0.5, 0.75, 1]
samples per symbol	40
symbol span	10
total number of coefficients	401

**Table 3:** Simulation scenarios.

Simulation Number	Modulated Bands	Rx g(t) Type	Tx p(t) Type
1	1	Bessel	Raised cosine
2	1		Matched Bessel
3	1	Butterworth	Raised cosine
4	1		Matched Butterworth
5	10	Bessel	Raised cosine
6	10		Matched Bessel
7	10	Butterworth	Raised cosine
8	10		Matched Butterworth
9	5 (odd)	Bessel	Raised cosine
10	5 (odd)		Matched Bessel
11	5 (odd)	Butterworth	Raised cosine
12	5 (odd)		Matched Butterworth

## Data Availability

Not applicable.
